# Personality Characteristics Determine Health-Related Quality of Life as an Outcome Indicator of Geriatric Inpatient Rehabilitation

**DOI:** 10.1155/2008/474618

**Published:** 2008-08-19

**Authors:** Jörg Richter, Martina Schwarz, Barbara Bauer

**Affiliations:** ^1^Centre for Child and Adolescent Mental Health, Eastern and Southern Region, 0405 Oslo, Norway; ^2^Bethesda Clinic, Geriatric Rehabilitation Hospital, Neubrandenburg, Germany

## Abstract

*Background*. The aim of the present study was to investigate the relationships between personality and quality of life during the course of geriatric rehabilitation, against the background of Cloninger's biosocial theory of personality. *Methods*. All consecutive patients of a geriatric rehabilitation clinic during one year were evaluated at admission and discharge (N = 687) by means of the ‘‘Vienna List’’ (a newly developed questionnaire for the assessment of quality of life in patients with severe dementia), and two variants of the Temperament and Character Inventory. *Results*. Self-directedness showed the most general and highest impact on quality of life and successful rehabilitation. *Conclusions*. It is probable in old and very old individuals who are on their highest level of maturity that the character represents the most important regulatory system in the encounter with challenges of daily life, which necessitates rehabilitation.

## 1. Introduction

The aim of geriatric rehabilitation is to improve the health conditions of the elderly and to maximise functions, mobility, and independence of elderly individuals following the impact of disease and/or injury, in order to improve their quality of life.

Quality of life (QoL) is an important outcome variable when the value of geriatric rehabilitation is evaluated [1, 2]. QoL is commonly defined as the perception by individuals of their position in life, in the context of the culture and value systems in which they live and in relation to their goals, expectation standards, and concerns. However, the definition of QoL remains vague and varies widely between research groups. Additionally, there is still a lack of well-adjusted assessment methods. There are various approaches to the conceptual structure of QoL in elderly persons. Marcoen et al. [3] presented a six-dimensional relational model of subjective well-being comprising psychological, physical, social, material, cultural, and existential aspects. Silberfeld et al. [4] described six groups of attributes of QoL of dementia patients: global impression of QoL, physical well-being, social/family well-being, emotional well-being, functional well-being, and cognition, whereas Rabins et al. [5] used five different assessment categories: social interaction, awareness of self, enjoyment of activities, feelings and mood, and response to surroundings. In very old and severely demented patients, Porzsolt et al. [6] empirically derived five important factors in the evaluation of QoL as communication abilities, aggression, and expression of negative feelings, mobility, and possibilities of physical contact.

QoL should be used as an outcome parameter of geriatric rehabilitation since it reflects major areas of rehabilitation goals in terms of the improvement in self-service, mobility, interpersonal behaviour, and communication.

To be able to address the global goal of rehabilitation of old and very old persons, that is, to improve their quality of life effectively, consistently, and adequately, knowledge is needed about determinants of quality of life. Quality of life is, of course, predominantly determined by the individuals' health conditions, including the sensory system and cognitive functions [7, 8], their functional level in daily life, coping recourses and available social support, their financial situation, environmental and community conditions, and last but not least, by the individual's personality.

Several investigations were performed with different approaches. State anxiety and behavioural coping, played no predictive role at admission into geriatric rehabilitation and became the strongest predictors of autonomy at discharge [9]. Extrovert personality measured by Eysenck's Personality Inventory and active coping strategy predicted improved ADL functions among former stroke patients at the three years follow-up stage [10]. Vigour alone was a predictor of current quality of life, while emotional stability was related to psychological well-being and satisfaction with significant relationships being reported by a Swedish study in 100 elderly persons by Hagberg et al. [11] using the Gordon Personality Inventory [12]. Ascendancy and ability to maintain personal relations were related to an optimistic outlook on life and an absence of psychosomatic symptoms. Summarising their results, the authors concluded that various personality characteristics are related to various quality of life dimensions. However, comparison of the investigations is complicated by the differing theoretical approaches and measures.

Recently, Cloninger proposed a psychobiological theory of personality including the behavioural systems of temperament and character, which are assumed to be related to two major neural systems for the adaptation to experiences at various levels [13–15]. The temperament dimensions: novelty seeking (tendency towards exhilaration in response to novel stimuli or cues), harm avoidance (bias in the inhibition or cessation of behaviour), reward dependence (tendency to maintain or pursue ongoing behaviour), and persistence (perseverance in behaviour despite frustration and fatigue) are defined as genetically homogenous and independently inherited and reflecting individual differences in conditioned emotional responses, such as anger, fear, love, and tenacity. The character dimensions self-directedness, cooperativeness, and self-transcendence are defined as reflecting individual differences in self-concepts according to the extent of identification with themselves as autonomous individual, with the humanity and with the whole universe. Based on this theory, Hansson et al. found lower levels of harm avoidance and higher levels of self-directedness to be significantly related to a better subjective quality of life in schizophrenic patients [16]. Self-directedness was significantly associated with a better subjective quality in all aspects measured, and explained a substantial amount of variance (range of 4–12%).

Therefore, the aim of our present study was the application of the biosocial theory of personality by Cloninger [14, 15] with respect to its major personality domains such as temperament and character when investigating the relationships between personality characteristics and quality of life during the course of geriatric rehabilitation. This project met several challenges. An extensive literature search yielded neither a measurement for personality nor for quality of life which could be easily used in the population of severely disturbed old and very old patients and which would be likely to be of high specificity and sensitivity to changes during the course of rehabilitation.

Accordingly, our research questions were the following: are personality characteristics in terms of temperament and character determinants of the outcome in geriatric rehabilitation? Is it possible to demonstrate distinct relationships between particular domains of quality of life, which are important in the context of geriatric rehabilitation including, for example, communication abilities, mobility, and daily functioning, with a particular pattern of personality variables in the population of interest?

More specifically, we hypothesised that

- self-directedness is related to all measured aspects of quality of life [16];
- cooperativeness and reward dependence predominantly influence aspects relating to social interaction like the expression of aggressive or other negative emotions and physical contact;
- novelty seeking and persistence are involved in communication and mobility capabilities; and
- harm avoidance combined with self-directedness is assumed to be related to emotional aspects as suggested by the ample evidence in the literature concerning their relationships with depression [17–19].

## 2. Methods

Within the framework of a comprehensive research project, all consecutive patients of the Geriatric Rehabilitation Clinic in Neubrandenburg (Neubrandenburg, Germany) being admitted from November 2001 to October 2002 were evaluated at admission and discharge [20, 21]. Six hundred and eighty seven patients were contacted at their admission (see Table 1). Four of them died in the clinic. Due to their poor health status at admission, all patients provided a written informed consent to participating in the project at discharge when their health condition had improved. The research protocol was approved by the Ethical Review Committee of Mecklenburg-Vorpommern.

The physician conducted a comprehensive evaluation of all patients on the day of their admission. This evaluation focussed upon the assessment of the functional deficits and the patient's social situation. Individual treatment goals and treatments were derived from the results of the evaluation. Treatment programmes were tailored to meet the needs of the patient. The nurses set out goals specific to the individual and care plans based on the care concepts of Roper [22–24] and Bobath [25–28]. Dependent on the condition of the patient, physiotherapeutic, occupational-therapeutic, speech therapeutic, and psychotherapeutic treatments were instigated in addition to the use of pharmaceuticals. The professionals predominantly performed the treatment in individual sessions. Unfortunately, the time of inpatient rehabilitation in Germany is generally limited to three weeks due to the restrictions set by the health insurances companies.

The rehabilitation diagnoses were performed following the criteria of ICD-10. Age, gender, and the duration of the acute inpatient treatment before rehabilitation were used in the present analyses.

Personality in terms of temperament and character was measured based on Cloninger's biosocial theory of personality by means of two methods that were completed by different proxy raters. An abbreviated version of the Temperament and Character Interview [29] was performed by one of the authors with the patients at discharge. Four hundred and ninety nine patients could be interviewed. The patients who could be interviewed received higher scores in the Mini Mental State Examination at admission (*t* = 4.52; *P* < .001; 24.2 ± 4.2 versus 21.9 ± 5.2) and more comorbid diagnosis (*t* = 3.9; *P* < .001) than those who could not be interviewed. The instrument consists of 25 questions, one each for every original subscale of the personality dimensions of the Temperament and Character Inventory. The interview is highly structured with clearly defined answers and needs about 30 minutes to be completed. It was included in the closing talk at discharge. The selection of the questions was oriented towards the life of elderly individuals. A score for each dimension (novelty seeking, harm avoidance, reward dependence, persistence, self-directedness, cooperativeness, and self-transcendence) was calculated by summing up the scores within each dimension divided by the number of items in the dimension, which enables a more direct comparison of the dimensions.

**Sample Items**

Are you usually more worried than most people that something might go wrong in the future?

- Undecided.
- If “yes”—how typical is it for you that you are worried that something might go wrong in the future?—“Highly typical” or “typical.”
- If “no”—are you usually less worried that something might go wrong in the future?—“Yes” or “no.”

Are you more likely to cry at a sad movie than most people?

- Undecided.
- If “yes”—do you cry at every sad movie? “Yes” or “no.”
- If “no”—did you ever cry at a sad movie? “Yes” or “no.”

**Figure 1:**
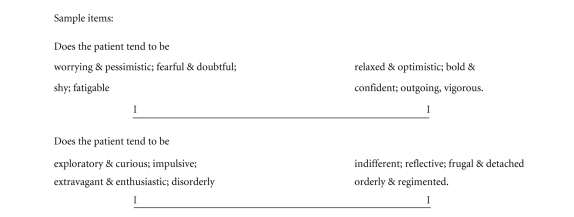


Additionally, the physiotherapist was trained to complete a visual analogue scale (VAS) relating to each personality dimension with descriptions of the anchor points for high and low expressions on the dimension scale (see Figure 1).

The physiotherapist was used as proxy rater because of her frequent and intensive contact with the patients, evaluating 612 of the patients during the investigation period. Both subsamples investigated (TCI interview and VAS) were representative of the total patient population during one year without systematic dropouts in any of the diagnostic groups. The modification of the methods was necessary in order to limit the strain placed on the old and very old patients during the interview.

A questionnaire for the assessment of quality of life in dementia patients, the “Vienna List” by Porzsolt et al. [6, 20, 21], was completed for each patient by specially trained nurses as proxy ratings at admission and discharge. This list consists of 41 items subdivided between five factors: communication (15 items), negative affect (10 items), physical contact (5 items), aggression (4 items), and mobility (6 items) which have to be rated on a 5-point scale from 1 = never to 5 = always. Cronbach's alpha coefficients as a measure of internal consistency between 0.93 for factor “communication” and 0.81 for “mobility” were calculated for nurses' ratings. This instrument was chosen for two reasons: (a) many of the patients in geriatric rehabilitation suffer at admission from functional rather than cognitive deficits, which are highly comparable with those suffered by severely demented patients, and (b) to demonstrate its specificity concerning rehabilitation diagnosis and the related functional disturbances, as well as its sensitivity to changes in functional status [20, 21].

**Statistics**

Mean scores, standard deviations, and frequencies were calculated. Pearson correlation coefficients of quality of life scores from discharge and of the relative changes of the quality of life scores from admission to discharge with personality variables were calculated and controlled for the influence of age and gender. Multivariate analyses of variance were calculated with the personality scores and with quality of life variables as dependent, gender as fixed factor, and age and duration of acute inpatient care before rehabilitation as covariates. Multiple regression analyses (method: enter) were performed with the personality scores as independent and the quality of life scores as dependent variables. The SPSS software was used.

## 3. Results

Most of the patients belonged to the group suffering from various fractures, followed by a group of patients suffering from stroke or other brain injuries (see Table 1). A significant relationship between gender and patients group occurred (*χ*2 = 33.9; *df* = 6; *P* < .001). On average, females suffered more often from hip or limb fractures, whereas males suffered more often from stroke and other brain injuries, abdominal and kidney disease, and from peripheral circulatory diseases.

The results of the MANOVA concerning QoL factors from admission showed significant main effects for gender (Pillai's trace = 0.04; *F*(5/650) = 4.32; *P* = .001; *η* ^2^ = 0.04) based on differences in the factors aggression and physical contact and for the duration of acute care (Pillai's trace = 0.03; *F*(5/650) = 3.14; *P* = .008; *η* ^2^ = 0.03) based on differences in factors communication abilities and negative affect (see Table 2 for descriptors). There was neither a significant main effect for age nor any significant interaction between one of the independent variables. With regard to discharge quality of life scores, significant main effects occurred for gender (Pillai's trace = 0.09; *F*(5/650) = 10.70; *P* < .001; *η* ^2^ = 0.08) based on differences in factors communication abilities, aggression and negative affect, for the duration of acute care (Pillai's trace = 0.03; *F*(5/650) = 2.87; *P* = .014; *η* ^2^ = 0.03) based on differences in factors communication abilities, aggression and mobility, and for age (Pillai's trace = 0.03; *F*(5/650) = 2.98; *P* = .011; *η* ^2^ = 0.03). Again, interaction terms were not significant.

When searching for differences in personality, significant main effects were found for gender (Pillai's trace = 0.03; *F*(7/678) = 2.16; *P* = .037; *η* ^2^ = 0.03) based on differences in harm avoidance, cooperativeness, and self-transcendence, and for age (Pillai's trace = 0.06; *F*(7/678) = 4.40; *P* < .001; *η* ^2^ = 0.06) based on differences in novelty seeking and reward dependence when using the results of the TCI interview (see Table 3 for descriptives). Concerning the visual analogue scales, only a significant main effect for gender (Pillai's trace = 0.03; *F*(7/678) = 2.54; *P* = .014; *η* ^2^ = 0.03) based on differences in harm avoidance and novelty seeking could be found.

When the relationships between quality of life and personality scores were controlled for the influence of age and gender, significant correlation coefficients were most pronounced for the assessment at discharge, for communication abilities, aggression, and negative affect, for self-directedness, and with higher coefficients for the relationships with scores of the visual analogue scales compared to the interview data (see Table 4). Communication abilities were related to self-directedness and negatively to harm avoidance (interview) and to all personality scores of the Visual Analogue Scales. The higher reward dependence and persistence were rated by the physiotherapist, the greater the evaluated differences in communication abilities between admission and discharge.

The more self-directed and cooperative (interview) the patients were, the less aggressive they were perceived to be at discharge, and the more cooperative and self-transcendent they were rated (VAS). The more cooperative, the more self-transcendent, and the more dependent on rewards the patients were (VAS), the less they behaved aggressively.

The higher the scores on self-directedness (interview), cooperativeness and persistence; and the lower that for harm avoidance (VAS) were, the more mobile the patients were evaluated to be at admission.

The more self-directed; less harm avoidant (interview); more reward dependent, persistent, and cooperative the patients were (VAS), the less negative affect was observed at discharge. The personality measures' scores were able to explain substantial amounts of variance in all five quality-of-life scores at discharge as well as of the relative changes during rehabilitation in communication, aggression, and negative affect, with varying contributions from the several temperament and character dimensions (see Table 5). Whereas reward dependence is exclusively responsible for the significant results (interview and VAS) concerning physical contact, harm avoidance, and self-directedness were the significant factors in the regression equations for negative affect. Self-directedness (interview) and harm avoidance were responsible for the significant results related to mobility. Whereas self-directedness and novelty seeking (interview) and, additionally, cooperativeness and persistence (VAS) contributed substantially to the prediction of the communication abilities at discharge, reward dependence (interview and VAS) remained as significant in the equation for the prediction of the changes in communication abilities combined with self-directedness for the interview data only. Aggression at admission could be predicted by cooperativeness scores combined with the scores of self-directedness and harm avoidance (interview) and novelty seeking, persistence, and self-transcendence (VAS), respectively, and the changes in the expression of aggression were substantially explained by self-directedness (interview) and by novelty seeking and self-transcendence (VAS).

## 4. Discussion

The aim of the present investigations was to evaluate the relationships between personality characteristics and quality of life in the course of geriatric rehabilitation, independent of the patients' particular illnesses. It was theoretically based on the biosocial theory of personality [13–15] with its subdivision of personality in temperament and character and on a model of quality of life by Porzsolt et al. [6, 20, 21] relating to very old and severely demented patients. There are only a few studies in the literature concerning the topic, each using different theoretical approaches and measurements.

A large representative sample of patients who were treated during one year at the rehabilitation clinic was included in the study. Limitations of the interpretations of the results are predominantly due to the “soft” measurements relating to personality characteristics, which were used for the first time with the presented design, even though they are based on a well-established theoretical background.

However, we found substantial results in terms of significant correlation and regression coefficients, which are in line with the theoretical background and supported our hypotheses even though most of the coefficients were of small to medium effect size. That was expected as personality characteristics are only one of the determinants of quality of life. Of course, health conditions and the patient's functional status represent the major influential factors relating to quality of life in the course of geriatric rehabilitation.

The gender differences identified relating to quality of life scores and to personality partly represent gender-related social stereotypes in terms of men being more likely to speak loudly (aggression) and being women rather more focused on physical contact and being more frequently described as becoming sad or depressed (negative affect). Furthermore, lower levels of novelty seeking and higher harm avoidance and cooperativeness were found in women compared to men in several studies, as was the age dependency of novelty seeking and reward dependence [30, 31].

There were various differences in correlations and regression results found depending upon the measurement of personality—interview versus VAS—in terms of higher scores derived for VAS the data mostly related to the communication, aggression, and negative affect factors. These differences are probably not exclusively due to the different source of information—structured self-description versus proxy rating. The physiotherapist was heavily involved in the treatment process of the patients, with physiotherapy often being perceived as hard work involving several conflicts between the demands of the therapist and the actual abilities and/or willingness to follow them on the part of the patient. This close relationship might have caused a bias in the evaluation of personality characteristics, which is reflected by the dominance concerning relationships with the factors communication, aggression, and negative affect. The higher importance of cooperativeness in these relationships based on VAS compared to interview data would support the presented explanation.

Self-directedness proved to be of substantial impact referred to all measured domains of quality of life in the course of geriatric rehabilitation. Mature, responsible, reliable, and well-integrated individuals are more engaged in their own rehabilitation, which causes better communication and mobility abilities, less aggression and negative affect compared to individuals low in self-directedness. This corresponds to the results in the literature reported by Hansson et al. [16] who found self-directedness to be significantly associated with all measured aspects of well-being explaining 4 to 12% of variance. In our study, we found most of the determination coefficients to be on a comparable level concerning the prediction of changes and higher levels for the prediction of outcome referred to communication, aggression, and negative affect.

Communication and mobility factors reflecting a wide range of abilities and personality characteristics were accordingly found to be related to several personality dimensions—cooperativeness (VAS data), novelty seeking and harm avoidance were affected. The combination of these two temperament dimensions might have caused approach (high novelty seeking)—avoidance (high harm avoidance) conflicts in some patients in the course of rehabilitation including, for example, relearning mobility abilities despite pain and lack of physical strength. However, a mature, developed character in terms of high self-directedness and high cooperativeness might enable individuals to cope with these contradictions which are partly caused by temperament make-up. However, the fact that change in communication is predominantly predictable by reward dependence points to the dependency of changes during rehabilitation upon intensive and effective emotional rewards for even small steps of progress being provided by all involved staff.

Interestingly, physical contact was exclusively related to the temperament dimension reward dependence implying that the more dependent on emotional rewards the patient is, the more easily she/he is able to handle physical contact.

Negative affect was related to self-directedness and harm avoidance based on both measures, reflecting the well-established importance of these two personality dimensions for depressive mood and their sensibility to changes of depressive mood [17, 19].

## 5. Conclusions

In conclusion, we were able to confirm our hypotheses and the application of the biosocial theory of personality to the determination of quality of life in the course of geriatric rehabilitation was successful, leading to some differentiating results in line with the theoretical background and clinical practice. Self-directedness was established as the personality dimension with the most general and highest impact on quality of life and successful rehabilitation. It appears that character dimensions in terms of self-directedness and cooperativeness are of higher importance than temperament dimensions reflected by higher correlation coefficients. It is likely that in old and very old individuals, who are at their highest level of maturity, the character represents the most important regulatory system when individuals are confronted with the challenges of daily life including severe disturbances in health and functional conditions, which caused the necessity of rehabilitation in old and very old individuals.

Furthermore, it can be concluded that consideration of the personality characteristics of geriatric rehabilitation patients can improve the effectiveness of the rehabilitation process which, in turn, can improve the quality of life of the patients. For example, highly reward dependent individuals should be continuously positively reinforced [32]; whilst the treatment of patients who are high novelty seekers should be process-oriented, without the setting of clearly defined targets, in order to encourage their own activity. On the other hand, low novelty seekers require clearly defined targets from the rehabilitation team; and highly self-directed individuals can be guided to a more autonomous rehabilitation in contrast to low self-directed patients who are in need of more frequent contact with the therapists who need to motivate the patients to achieve their targets and complete their rehabilitation.

## Figures and Tables

**Table 1:** Characteristic of the patients.

	Male	Female	Total
Number N/%	188/27.4	499/72.6	687
Age in years (*x* ± SD)	74.5 ± 8.6	78.9 ± 7.6	77.7 ± 8.1
Time of acute inpatient care before rehabilitation in days (*x* ± SD)	27.4 ± 22.9	22.4 ± 14.3	23.7 ± 17.1
Time of inpatient rehabilitation in days (*x* ± SD)	21.3 ± 6.5	21.5 ± 6.6	21.5 ± 6.6
Number of comorbid diagnosis	5.4 ± 2.5	4.9 ± 2.1	5.0 ± 2.2
Range	0–14	0–14	0–14
Heart and lung diseases	29/15.4	55/11.0	84/12.2
Stroke and other brain diseases	68/36.2	127/25.5	195/28.4
Fractures	31/17.0	199/39.9	231/33.6
Extrapyramidal and walking diseases	17/9.0	37/7.4	54/7.9
Abdominal and kidney diseases	20/10.6	39/7.8	59/8.6
Peripheral circulatory diseases	15/8.0	22/4.4	37/5.4
Others	7/3.7	20/4.0	27/3.9

**Table 2:** Means scores of QoL scores from both assessments by gender.

	Male	Female	Total
Communication—admission	3.7 ± 0.6	3.8 ± 0.6	3.7 ± 0.6
Communication—discharge	3.7 ± 0.6	3.9 ± 0.6	3.9 ± 0.6
Aggression—admission	1.3 ± 0.5	1.2 ± 0.4	1.2 ± 0.4
Aggression—discharge	1.4 ± 0.6	1.2 ± 0.5	1.3 ± 0.5
Negative affect—admission	1.7 ± 0.5	1.8 ± 0.6	1.8 ± 0.6
Negative affect—discharge	1.7 ± 0.5	1.9 ± 0.6	1.8 ± 0.6
Physical contact—admission	4.2 ± 0.9	4.4 ± 0.8	4.3 ± 0.8
Physical contact—discharge	4.3 ± 0.9	4.4 ± 0.8	4.4 ± 0.8
Mobility—admission	2.1 ± 0.5	2.1 ± 0.5	2.1 ± 0.5
Mobility—discharge	2.3 ± 0.4	2.3 ± 0.4	2.3 ± 0.4

**Table 3:** Mean scores of both personality measures.

	TCI interview		TCI analogue scales	
	Male	Female	Male	Female
Novelty seeking	2.4 ± 0.7	2.3 ± 0.6	2.9 ± 1.4	2.6 ± 1.3
Harm avoidance	2.5 ± 0.8	2.9 ± 0.9	2.9 ± 1.6	3.2 ± 1.5
Reward dependence	2.6 ± 0.8	2.8 ± 0.8	3.7 ± 1.1	3.7 ± 1.1
Persistence	3.8 ± 1.1	3.6 ± 1.1	3.7 ± 1.4	3.5 ± 1.3
Self-directedness	4.4 ± 0.6	4.3 ± 0.6	3.5 ± 1.3	3.3 ± 1.3
Cooperativeness	3.4 ± 0.6	3.6 ± 0.6	3.6 ± 1.2	3.7 ± 1.2
Self-transcendence	2.4 ± 1.0	2.1 ± 1.0	3.7 ± 1.1	3.9 ± 1.0

**Table 4:** Partial correlation coefficients between personality (TCI interview—first row; TCI visual analogue scales—second row) and quality-of-life variables (controlled for age and gender).

Group	NS	HA	RD	PS	SD	CO	ST
Communication							
Discharge	0.11*	−0.14**	−0.02	0.13**	0.26***	0.08	0.13**
	0.27***	−0.25***	0.32***	0.45***	0.42***	0.38***	0.23***
Difference	−0.02	0.003	−0.01	−0.0003	−0.03	0.01	0.02
	−.01	0.03	−0.16***	−0.15***	−0.09*	−0.11**	−0.13***

Aggression							
Discharge	0.07	0.005	−0.03	−0.06	−0.30***	−0.17***	−0.03
	0.10*	0.12**	−0.27***	−0.25***	−0.23***	−0.32***	−0.35***
Difference	−0.04	0.007	0.04	0.04	0.15***	0.11*	0.05
	−0.06	0.06	0.16***	0.10**	0.12**	0.19***	0.20***

Physical contact admission							
Discharge	0.05	0.03	0.03	0.04	0.06	0.02	0.07
	0.02	−0.004	0.17***	0.10*	0.05	0.12**	0.15***
Difference	−0.09	−0.007	−0.04	−0.01	0.005	0.01	−0.06
	−0.03	−0.04	−0.14***	−0.08	−0.05	−0.08	−0.09*

Mobility							
Discharge	0.09*	−0.09*	−0.06	0.13**	0.20***	0.04	0.10*
	0.16***	−0.21***	0.09*	0.20***	0.21***	0.19***	0.09*
Difference	−0.02	−0.002	0.04	−0.01	−0.10*	0.07	−0.05
	−0.05	−0.002	0.07	−0.05	−0.09*	0.09*	−0.02

Negative affect							
Discharge	−0.04	0.16***	−0.02	−0.09*	−0.19***	−0.09*	−0.04
	−0.10*	0.32***	−0.21***	−0.31***	−0.32***	−0.31***	0.22***
Difference	0.003	−0.05	0.06	0.0007	0.07	0.11*	−0.03
	0.01	−0.08	0.04	0.06	0.05	0.04	0.0003

**Table 5:** Multivariate regression analysis with QoL variables and their changes as dependent and personality (interview-data—1st row; visual analogue scale—2nd row) as independent.

Group	Adjusted *r* ^2^	*F*	*P*
Communication			
Discharge	0.24	26.92	<.001
	0.25	28.12	<.001
Difference	0.03	2.53	.014
	0.04	3.20	.002

Aggression			
Discharge	0.18	18,11	<.001
	0,19	19,37	<.001
Difference	0,07	6,30	<.001
	0,07	6,69	<.001

Physical contact admission			
Discharge	0,04	3,75	.001
	0,04	3,87	<.001
Difference	0.008	0.65	.715
	0.02	1.87	.072

Mobility			
Discharge	0.07	6.48	<.001
	0.08	6.91	<.001
Difference	0.02	1.55	.149
	0.02	1.50	.166

Negative affect			
Discharge	0.18	18.41	<.001
	0.18	19.03	<.001
Difference	0.03	2.18	.034
	0.02	1.99	.054
